# Developmental ethanol exposure produces deficits in long‐term potentiation in vivo that persist following postnatal choline supplementation

**DOI:** 10.1111/acer.15384

**Published:** 2024-06-08

**Authors:** A. K. Titterness, E. L. Gräfe, C. Acosta, C. Rodriguez, J. D. Thomas, B. R. Christie

**Affiliations:** ^1^ Division of Medical Sciences University of Victoria Victoria British Columbia Canada; ^2^ Department of Psychology San Diego State University San Diego California USA; ^3^ Island Medical Program and Department of Cellular and Physiological Sciences University of British Columbia Victoria British Columbia Canada; ^4^ Institute for Aging and Life‐Long Health University of Victoria Victoria British Columbia Canada

**Keywords:** dentate gyrus, developmental ethanol exposure, postnatal choline supplementation, sex differences, synaptic plasticity

## Abstract

**Background:**

Fetal alcohol spectrum disorder (FASD) is one of the leading causes of neurodevelopmental disorder for which there is a pressing need for an effective treatment. Recent studies have investigated the essential nutrient choline as a postnatal treatment option. Supplementation with choline has produced improvements in behavioral tasks related to learning and memory and reverted changes in methylation signature following third‐trimester equivalent ethanol exposure. We examined whether there are related improvements in hippocampal synaptic plasticity in vivo.

**Methods:**

Sprague–Dawley offspring were administered binge‐levels of ethanol from postnatal day (PND) 4 to 9, then treated with choline chloride (100 mg/kg/day) from PND 10 to 30. In vivo electrophysiology was performed on male and female offspring from PND 55 to 70. Long‐term potentiation (LTP) was induced in the medial perforant pathway of the dentate gyrus using a theta‐burst stimulation (TBS) protocol, and field‐evoked postsynaptic potentials (EPSPs) were evoked for 60 min following the conditioning stimulus.

**Results:**

Developmental ethanol exposure caused long‐lasting deficits in LTP of the slope of the evoked responses and in the amplitude of the population spike potentiation. Neither deficit was rescued by postnatal choline supplementation.

**Conclusions:**

In contrast to our prior findings that choline can improve hippocampal plasticity (*Nutrients*, 2022, 14, 2004), here we found that deficits in hippocampal synaptic plasticity due to developmental ethanol exposure persisted into adulthood despite adolescent choline supplementation. Future research should examine more subtle changes in synaptic plasticity to identify synaptic changes that mirror behavioral improvements.

## Graphical abstract

This work found that third‐trimester ethanol exposure produced long‐lasting deficits in hippocampal synaptic plasticity in adult animals (greater than postnatal day 50) that were not rescued by postnatal choline supplementation early in life (postnatal day 10–30). These results indicate that longer periods of supplementation may be required to produce more persistent benefits.

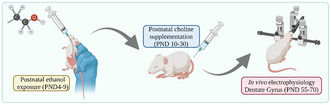

## INTRODUCTION

Developmental ethanol (ETOH) exposure induces a broad range of birth defects and lifelong neurodevelopmental deficits that have been collectively termed fetal alcohol spectrum disorders (FASD). Despite widespread recognition of the potential for alcohol use during pregnancy to produce these harmful effects, FASD continues to affect between 2% and 5% of the population (May et al., 2014). The array of adverse effects resulting from exposure to ethanol in utero include a common pattern of facial dysmorphia, growth deficiencies, neuroanatomical abnormalities, and central nervous system dysfunction (del Campo & Jones, 2017; Riley et al., 2011). Individuals with FASD also present with impairments in hippocampal‐dependent learning and memory (Mattson et al., 2011, 2019). The hippocampus is a structure that is intimately involved in learning and memory and is particularly vulnerable to insults following the developmental ETOH exposure (Berman & Hannigan, 2000). ETOH impairs hippocampal‐dependent learning and memory, alters structural morphology (Berman & Hannigan, 2000; Hamilton et al., 2011; Klintsova et al., 2007; Livy et al., 2003), and alters neuronal connectivity and transmission (Berman & Hannigan, 2000; West & Hamre, 1985). In particular, developmental ETOH exposure can impair synaptic plasticity, a putative mechanism underlying learning and memory. Specifically, long‐term potentiation (LTP) and long‐term depression (LTD) are impaired across the lifespan in males following the developmental ETOH exposure (Fontaine et al., 2019; Kervern et al., 2015; Sickmann et al., 2014). In females, however, developmental ETOH exposure affects LTP and LTD in adolescence (Fontaine et al., 2019) but not adulthood (Sickmann et al., 2014; Titterness & Christie, 2012). Therefore, males and females are differentially affected by developmental ETOH exposure, suggesting that therapeutic interventions aimed at alleviating ETOH‐induced deficits might also differentially affect males and females.

While there is currently no specific treatment for FASD, one promising therapeutic candidate for improving cognitive and behavioral impairments in FASD is the essential nutrient choline. Although choline can be derived from de novo synthesis, choline levels largely depend on dietary intake (Garner et al., 1995). During pregnancy, the demand for choline increases and adequate choline intake has become an increasingly important recommendation for expectant mothers (Korsmo et al., 2019). Choline is involved in many processes, including phospholipid generation, acetylcholine synthesis, and methyl donation in the 1‐carbon metabolism cycle (Zeisel, 2011); therefore, choline can have a substantial impact on the developing hippocampus. Prenatal choline supplementation affects hippocampal pyramidal cell morphology (Li et al., 2004), increases neurogenesis (Albright et al., 1999; Craciunescu et al., 2003), enhances cholinergic functioning, and decreases LTP thresholds (Li et al., 2004; Mellott et al., 2004; Pyapali et al., 1998). Some of these effects are long‐lasting; choline induces hippocampal neurogenesis that persists into adulthood (Glenn et al., 2007) and protects the hippocampus from insults later in life (Blusztajn & Mellott, 2013).

There are many preclinical studies demonstrating that choline supplementation mitigates the neurodevelopmental disabilities associated with ETOH when administered either pre‐ and postnatally. In rodents, prenatal choline during pregnancy ameliorates the effects of alcohol on birth and brain weight, early motor development, working memory, spontaneous alternation (Thomas et al., 2009, 2010), and balance deficits (Bearer et al., 2015). When administered postnatally, choline reduces the severity of open‐field hyperactivity (Monk et al., 2012), trace fear conditioning (Wagner & Hunt, 2006), improves working memory, performance in discrimination tasks (Thomas et al., 2000), and rescues deficits in spatial learning (Ryan et al., 2008). Preclinical evidence provides a clear impetus for clinical trials investigating the effectiveness of choline in children with FASD. While only a limited number of clinical studies exist to date (review by Ernst et al., 2022), postnatal choline intake in children exposed to alcohol prenatally may improve neurodevelopmental outcomes (Ernst et al., 2022). These studies suggest choline supplementation may be a viable treatment for FASD; however, more studies are required to understand the mechanism and limitations of choline supplementation before it can be recommended as an effective treatment.

Due to its highly plastic nature, the hippocampus represents an important target for choline treatments following ETOH. Recently, we showed that prenatal ethanol exposure over the first two trimester equivalents decreased LTP in juvenile male, but not female, offspring, which was rescued with postnatal choline supplementation (Grafe et al., 2022). However, it is not clear whether third‐trimester equivalent ethanol exposure, which occurs postnatal in rodents, similarly reduces LTP and if choline has therapeutic potential in this model. Therefore, the objective of this study was to determine whether postnatal choline supplementation would result in long‐lasting benefits in hippocampal synaptic plasticity following the developmental ethanol exposure.

## METHODS

### Animal generation

Adult Sprague–Dawley breeders (Charles River Laboratories, Hollister, CA, USA) were maintained in a temperature‐ and humidity‐controlled mating colony of the SDSU Center for Behavioral Teratology. One adult male and one adult female were housed together overnight, and the presence of a seminal plug was used to indicate mating; this was denoted as gestational day (GD) 0. Once pregnant, dams were singly housed until they gave birth, which typically occurred on GD 22, also denoted as postnatal day (PND) 0. On PND 1, litters were culled to eight pups, maintaining four males and four females if possible. Pups were randomly assigned within litter to one of four treatment groups, in a 2 (Ethanol, Sham) × 2 (Choline, Vehicle) × 2 (male, female) design. No more than one sex pair from any litter was assigned to any treatment group to control for litter effects.

Offspring were exposed to ethanol from PND 4 to 9, a period equivalent to the third‐trimester brain growth spurt, as described previously (Ryan et al., 2008; Thomas et al., 2004, 2007). Ethanol (Sigma Aldrich, St. Louis, MO, USA) was delivered via oral intubation at a dose of 5.25 g/kg/day. The ethanol was dissolved in a milk diet (11.9% v/v) and administered in two intubations set 2 h apart each day, producing a binge‐like ethanol exposure. Two additional milk feedings were provided at 2‐h intervals each day, as intoxicated pups may not suckle well. Sham controls received four sham intubations each day, 2 h apart. Peak blood alcohol concentrations were determined on PND 6, 90 min after the second ethanol intubation. Twenty microliters of blood was collected via a tail clip, and plasma was separated and analyzed using the Analox Alcohol Analyzer (Model AM1; Analox, Instruments, Lunenberg, ME, USA). This paradigm produces blood alcohol concentrations between 320 and 410 mg/dL (Ryan et al., 2008; Thomas et al., 2004, 2007). Blood was collected from sham controls as well, but not analyzed. To ensure that investigators remained blind to treatment condition following exposure, pups' paws were tattooed with nontoxic veterinary tattoo ink (STONE Manufacturing and Supply Company, Kansas City, MO, USA) on PND 7.

From PND 10 to 30, half of the subjects from each exposure group were injected subcutaneously (s.c.) with 100 mg/kg/day of choline chloride (6.667 mL/kg/day; Balchem, New Hampton, NY, USA). Vehicle controls were injected with saline. Thus, there were four treatment groups: ethanol‐exposed (ETOH + S), ethanol‐exposed treated with choline (ETOH + C), choline treated only (CON + C), and controls (CON + S). Pups were weaned on PND 21 and separated by sex on PND 28. Previously, we have demonstrated that, while ETOH offspring may lag behind in weight gain, neither ETOH nor choline treatment altered weight gain long term as compared to artificially reared controls (Ryan et al., 2008; Thomas et al., 2004, 2007). All experimental procedures were approved by the San Diego State University (SDSU) Institutional Animal Care and Use Committee in accordance with the National Institutes of Health's *Guide for the Care and Use of Laboratory Animals*.

### In vivo electrophysiology

In vivo electrophysiology followed the same procedure as previously reported (Titterness & Christie, 2012). Briefly, male and female animals between PND 55 and 70 were anesthetized with urethane (1.5 g/kg, intraperitoneal) and placed in a Kopf stereotaxic apparatus. Body temperature was maintained at 37 ± 1°C using a grounded homoeothermic temperature control unit. A 125‐*μ*m‐diameter stainless steel recording electrode was placed into the hilus of the DG (3.5 mm posterior, 2.0 mm lateral to bregma) through a trephine hole. A 125‐*μ*m‐diameter monopolar stimulating electrode was placed in the medial perforant pathway (7.4 mm posterior, 3.0 mm lateral to bregma) and extracellular field potentials were recorded. The maximal response was determined, and the stimulation was set to generate a 1–2 mV population spike. Input/output curve was generated by stimulating at increasing pulse widths (0.04, 0.08, 0.12, 0.16, 0.20, and 0.24 ms in duration) at 0.067 Hz repeated five times. Input/output data are expressed as normalized values to the largest response. Prior to each experiment, a set of paired‐pulse stimuli (50‐ms interpulse interval; 0.12‐ms pulse width) were administered to verify that the stimulating electrodes were activating medial perforant path fibers as described previously (Christie & Abraham, 1994; Petersen et al., 2013). A stable baseline was then obtained by delivering single pulse stimulation (0.12‐ms pulse width; 0.067 Hz) for a minimum of 15 min. To induce LTP, a theta burst stimulation (TBS; four trains of 10 bursts of 5 pulses at 400 Hz with a 200‐ms interburst interval with 0.24‐ms pulse width) paradigm was delivered and then baseline stimulation (0.12‐ms pulse; 0.67 Hz) was resumed and recorded for 1 h. Following the postconditioning period, the animal was euthanized by an overdose of urethane to the brain. Extracellular field potentials were acquired using the custom written software (Lee Campbell; Getting Instruments, San Diego, CA, USA) and National Instruments data acquisition software. Signals were amplified and filtered at 1 and 3 Hz using a differential amplifier (Getting Instruments) and digitized at 5 Hz. Data are represented as a percent change from the average slope of the initial phase of the excitatory postsynaptic potential (EPSP; measured between 10% and 80% of the slope of the waveform) or percent change in the amplitude of the population spike.

### Statistical analysis

All data were analyzed using the JASP Software (Version 0.16.3). If there were no significant differences between data from male and female offspring, the data were combined. All male and female data are listed in Table S1. When assumptions of heterogeneity of variance (Levene's test *p* > 0.05) and normality were met (Shapiro–Wilkes test *p* > 0.05), data were analyzed with two‐way ANOVAs (Control, Ethanol) × (Saline, Choline). Tukey's post hoc test was used where appropriate. In LTP and population spike analysis, normality was not met (Shapiro–Wilkes test *p* < 0.05), and therefore, the data were transformed using a square root transformation before analysis. Input/output data were analyzed with a repeated‐measures ANOVA with a Greenhouse–Geissier correction for sphericity. Data are presented as average ± standard error of the mean (SEM). *p* < 0.05 was considered significant for all measures.

## RESULTS

### Choline treatment reduced evoked response excitability

**FIGURE 1:**
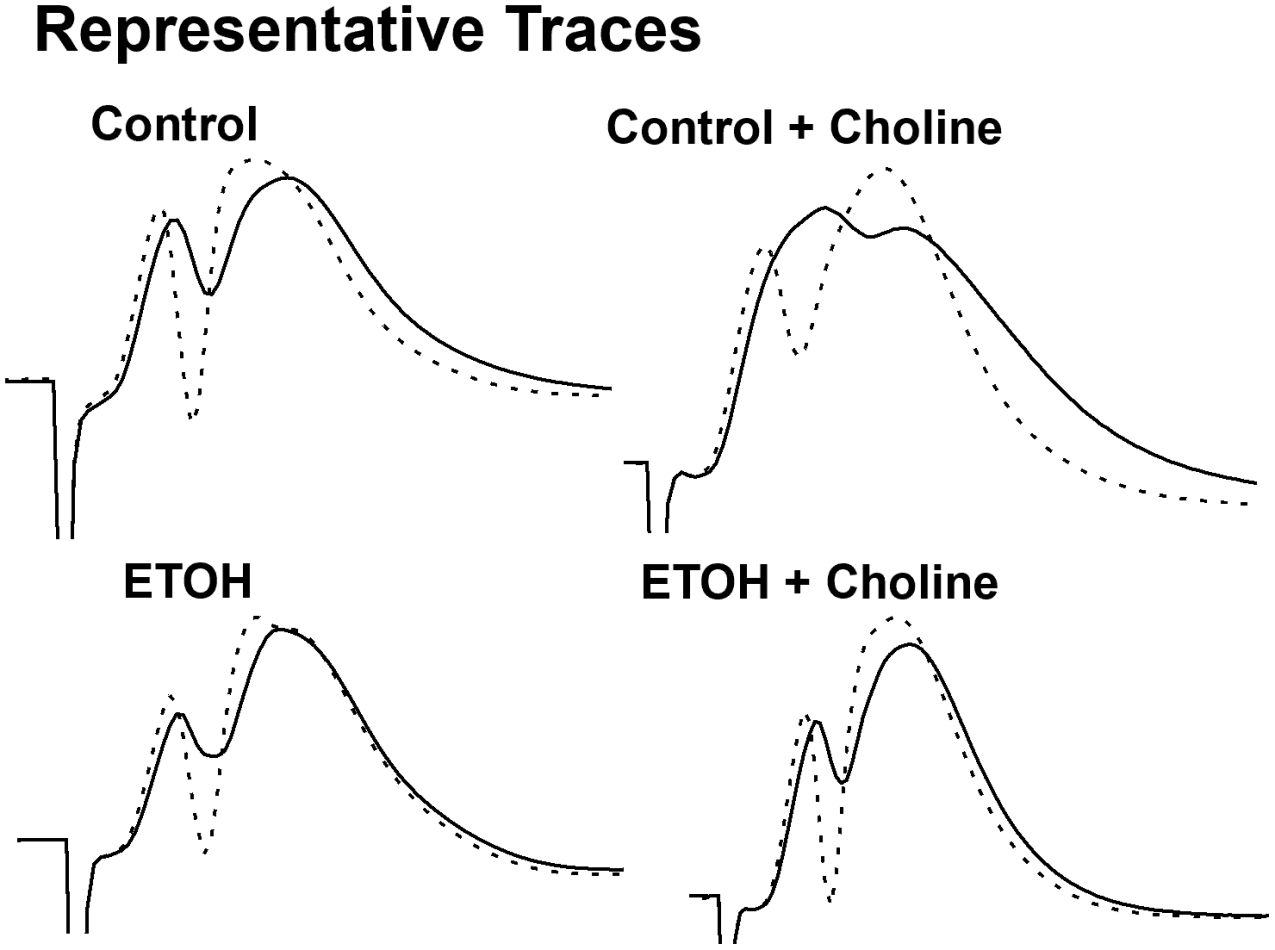
Representative traces. Example traces of MPP recordings before TBS (solid line) and after the 60 min postconditioning period (dotted line). ETOH: developmental ethanol exposure.

**FIGURE 2:**
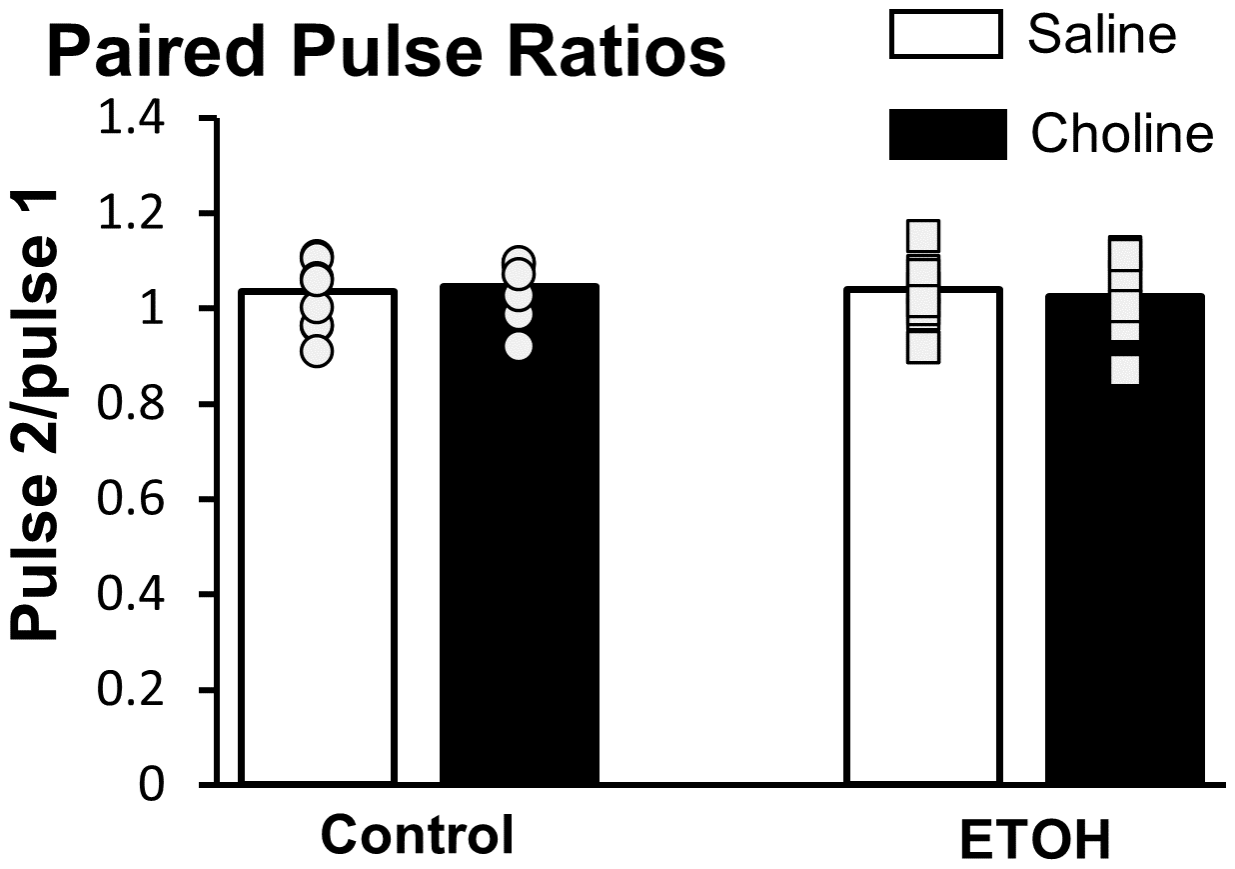
Paired‐pulse ratios are equivalent between conditions. Average paired‐pulse ratio (pulse 2/pulse 1) for Control and ETOH offspring. Bars are the average ratio for saline‐treated (white) and choline‐treated (black) offspring. Individual points represent the paired‐pulse ratio per animal. *N* = 14–19 animals per group. Error bars are ± SEM.

Field EPSPs (Figure 1) were reliably evoked by stimulating the medial perforant path input to the DG. To confirm the placement of the electrodes, a 50‐ms paired‐pulse stimuli was administered to ensure that minimal paired‐pulse facilitation was produced (Petersen et al., 2013) (Figure 2). There were no differences in the paired‐pulse ratios in any of the prenatal exposure groups (*F*_(1,59)_ = 0.20, *p* = 0.653, $\eta_{p}^{2}$ = 0.003), or for any of the postnatal treatments (*F*_(1,59)_ = 0.01, *p* = 0.942, $\eta_{p}^{2}$ = 0.00).

**FIGURE 3:**
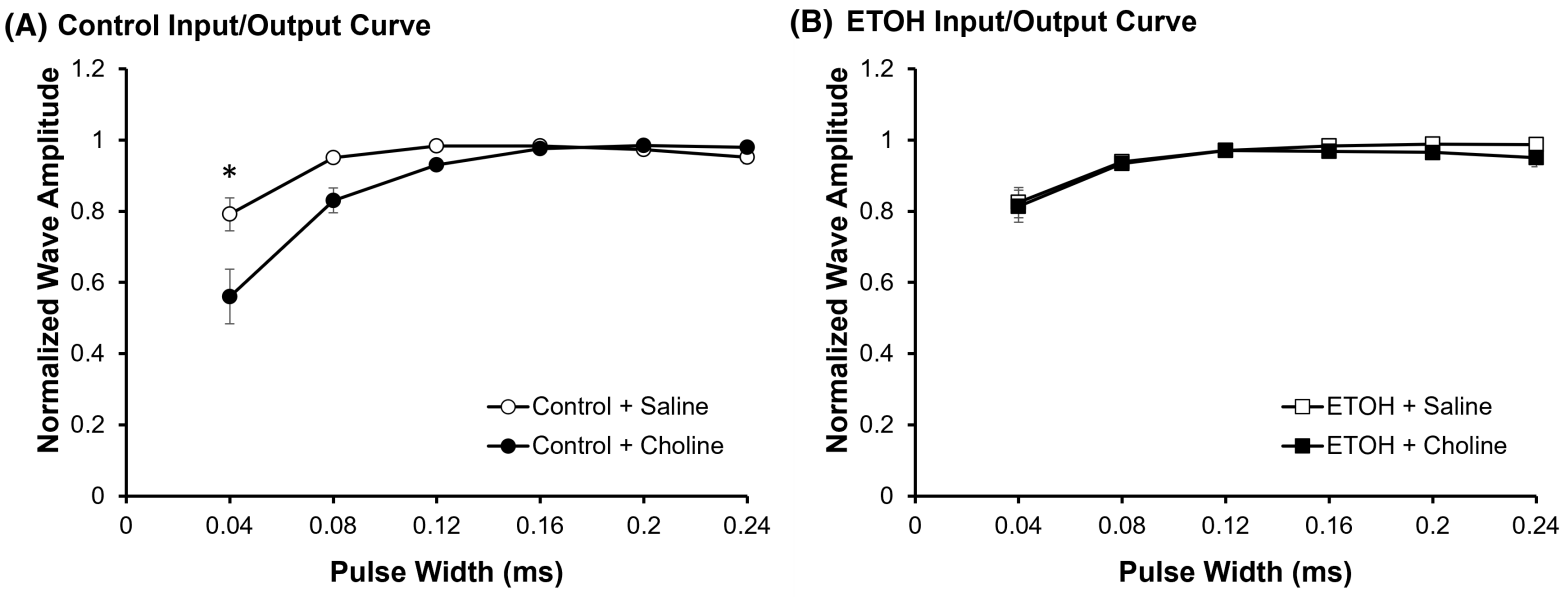
Choline reduced excitability at low pulse width input. Average normalized wave amplitude in Control (A) and ETOH (B) conditions, with saline‐treated (white) and choline‐treated (black) offspring. *p < 0.001. Error bars are ± SEM.

To determine stimulus–response relationships for the medial performant path synapses in the dentate gyrus, stimuli of increasing pulse widths (40–240 *μ*s) were given to examine the change in the EPSP slope (Figure 3A,B). As expected, for all groups, there was a significant increase in the normalized EPSP amplitude with increasing pulse width (*F*_(1.60,66.61)_ = 44.17, *p* < 0.001, $\eta_{p}^{2}$ = 0.469). An interaction was found between pulse width × ETOH × choline treatment (*F*_(1.33,66.61)_ = 3.864, *p* = 0.042, $\eta_{p}^{2}$ = 0.072). Post hoc analyses determined that there was a significant difference between CON + Choline group and all other groups at the smallest pulse width (0.04 ms; *p* < 0.001). Importantly, there were no differences in evoked responses at 120 *μ*s (*p* > 0.05), the width that was used for baseline stimulation with electrophysiology experiments.

### Choline does not rescue ethanol‐induced reduction in DG synaptic plasticity

**FIGURE 4:**
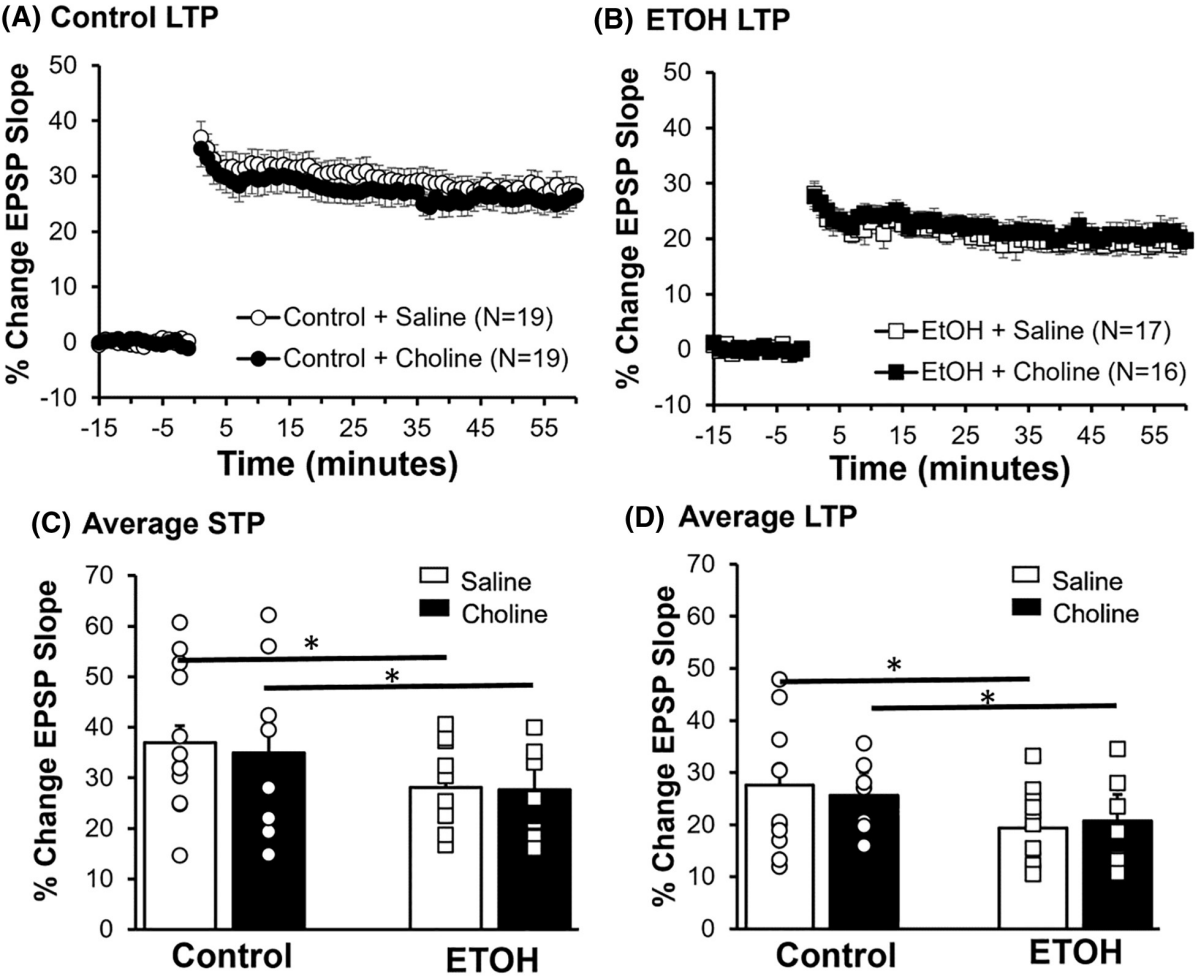
ETOH reduced short‐ and long‐term potentiation as compared to control offspring. Summary line graphs for Control (A) and ETOH (B), with saline‐treated offspring (white) and choline‐treated offspring (black). Average change in EPSP slope (minutes 0–60) is presented as a percent change from the preconditioning period (minutes −15 to 0). The average short‐term potentiation (STP), 1 min following TBS, (C) and long‐term potentiation (LTP), last 5 min of recording (D) are represented by the bars. Each point represents an individual animal. *p < 0.05. All error bars are ± SEM.

The effect of developmental ETOH exposure and choline treatment on synaptic plasticity was investigated next. An ANOVA found a significant main effect of ETOH on short‐term potentiation (STP: *F*_(1,67)_ = 8.62, *p* = 0.005, $\eta_{p}^{2}$ = 0.114) and LTP (*F*_(1,67)_ = 8.88, *p* = 0.004, $\eta_{p}^{2}$ = 0.117). Tukey's post hoc analyses revealed that STP in ETOH‐exposed animals was significantly less than controls (*t* = 2.93; *p* = 0.005). Furthermore, LTP in ETOH‐exposed animals was significantly less than controls (*t* = 2.98, *p* = 0.004). We then investigated whether choline treatment affected plasticity in control and ETOH‐exposed animals. An ANOVA found no effect of choline treatment on either STP (*F*_(1,67)_ = 0.21, *p* = 0.647, $\eta_{p}^{2}$ = 0.003) or LTP (LTP: *F*_(1,67)_ = 0.00, *p* = 0.995, $\eta_{p}^{2}$ = 0.00; Figure 4C,D).

**FIGURE 5:**
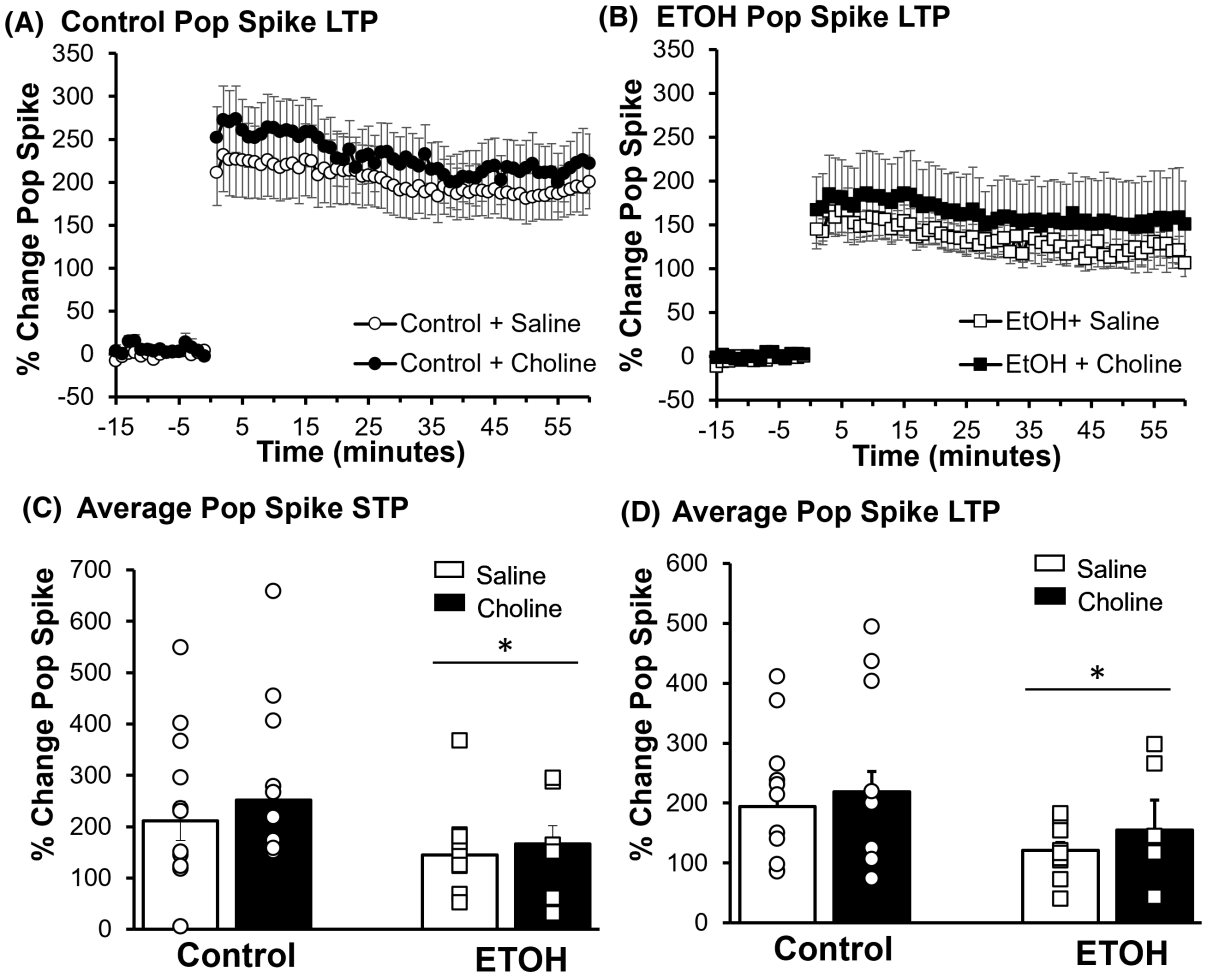
ETOH reduced short‐ and long‐term population spike potentiation. Summary traces for population (pop) spike potentiation for Control (A) and ETOH (B), with saline‐treated offspring (white) and choline‐treated offspring (black). Average change in population spike (minutes 0–60) is presented as a percent change from the preconditioning period (minutes −15 to 0). The average short‐term potentiation (STP), 1 min following TBS, (C) and long‐term potentiation (LTP), last 5 min of recording (D) are represented by the bars. Each point represents an individual animal. *p < 0.05. All error bars are ± SEM.

Changes in the degree of potentiation of the population spike were also examined. As shown in Figure 5A,B, an ANOVA found a main effect of diet on STP (*F*_(1,57)_ = 5.61, *p* = 0.021, $\eta_{p}^{2}$ = 0.090) and a Tukey's post hoc analysis found that the STP in the pop spike for ETOH‐exposed animals was significantly lower than controls (*t* = 2.37, *p* = 0.021). There was also a significant main effect of diet on LTP (*F*_(1,57)_ = 5.66, *p* = 0.021, $\eta_{p}^{2}$ = 0.092), and post hoc analyses revealed that LTP in ETOH‐exposed animals was significantly lower than controls (*t* = 2.56, *p* = 0.013). Choline treatment did not ameliorate these deficits in either control or choline groups (STP: *F*_(1,57)_ = 0.24, *p* = 0.629, $\eta_{p}^{2}$ = 0.004; Figure 5C. LTP: *F*_(1,57)_ = 0.11, *p* = 0.738, $\eta_{p}^{2}$ = 0.002). The results for the changes in STP and LTP for the population spike are summarized in Figure 5C,D, respectively.

## DISCUSSION

This work demonstrates for the first time that ETOH exposure during the third‐trimester equivalent produces deficits in both STP and LTP in the dentate gyrus in vivo. Surprisingly, choline administration did not rescue ETOH‐induced deficits to synaptic plasticity in the dentate gyrus. Additionally, basal sex differences were not observed between males and females. Taken together, these findings indicate that exposure to ethanol during the third‐trimester equivalent equally impaired DG LTP in males and females, an effect that was not ameliorated by choline administration from PND 10 to 30.

These findings add to the growing literature on the deleterious effects of developmental ETOH exposure on hippocampal synaptic plasticity. We have previously reported that exposure to ethanol during the gestational period reduces LTP in the hippocampus (Helfer et al., 2012; Sickmann et al., 2014; Titterness & Christie, 2012). The results of the current study extend these findings to the third‐trimester equivalent. It is important to note that the effects exposure to ETOH during development can be variable (Fontaine et al., 2016), and previous studies have shown that third‐trimester equivalent ETOH exposure did not reduce early adult LTP in either the CA1 (Bellinger et al., 1999) or the DG (Helfer et al., 2012; Patten et al., 2013). In particular, our findings are in contrast to Patten et al. (2013) who found that LTP in the DG was enhanced in females, but LTP was neither enhanced nor reduced in males following the equivalent ethanol exposure when examined during the prepubescent period. Sex‐specific effects of developmental ethanol exposure on hippocampal synaptic plasticity are not novel (Titterness & Christie, 2012), nor are basal sex differences in hippocampal synaptic plasticity (Gall et al., 2023; Safari et al., 2021; Titterness & Christie, 2012). However, sex differences may change with age. In fact, the lack of sex difference in the current study is in keeping with the work of others (Zitman & Richter‐Levin, 2013), who found that sex differences in DG LTP that are present during the prepubertal phase are absent in adulthood. Similar to the third‐trimester ethanol exposure, sex‐specific effects of gestational ETOH exposure on DG synaptic plasticity, similar to findings that effects of ETOH on hippocampal synaptic plasticity are also more likely to be uncovered prior to puberty, either immediately following exposure (Puglia & Valenzuela, 2010) or in early adolescence (Izumi et al., 2005). While we did not observe sex differences in the current study, the effect of estrogen on the observed synaptic plasticity cannot be ruled out. Within the DG, estrogen can reduce pop‐spike LTP and EPSP potentiation (Gupta et al., 2001). We did not track the stages of the estrous cycle for females used in the current study, so it is possible that the depressant effect of estrogen on DG LTP was inadvertently assessed in the current study. As there is mounting evidence of sexually dimorphic effects of developmental ETOH on hippocampal synaptic plasticity, it would be beneficial to thoroughly investigate the effect of endogenous estrogen on DG LTP to better understand some of the differences observed among studies. Gestational ETOH exposure (first two trimester equivalent) may also produce greater deficits in long‐term hippocampal synaptic plasticity, as deficits are evident for LTP in both the adult CA1 and DG (An & Zhang, 2013; Helfer et al., 2012; Sickmann et al., 2014; Swartzwelder et al., 1988; Varaschin et al., 2014) and deficits in long‐term depression (LTD) have also been reported (An & Zhang, 2013; Kervern et al., 2015).

In this study, we also found a long‐lasting decrease in the potentiation of the population spike with developmental ETOH. The population spike represents the activation of granule cells and the collective firing of action potentials. Following the conditioning stimuli that result in EPSP‐LTP, the population spike can also potentiate past what the increase in EPSP can explain alone. This phenomenon is known as E‐S potentiation. The mechanism of E‐S potentiation is thought to be due to changes in GABAergic transmission, as the application of picrotoxin, a GABA_A_ receptor antagonist, can occlude further E‐S potentiation (Chavez‐Noriega et al., 1989). In some cases, the potentiation of the population spike, and not potentiation of the EPSP, has been correlated with improved performance on learning and memory tasks (Kleschevnikov & Marchbanks, 1993). However, others have suggested that too much potentiation may lead to overactivation of granule cells and diminished spatial memory (Muellerleile et al., 2020). Our finding that ETOH reduced population spike potentiation is novel, as previously deficits in population spike amplitude potentiation have not been significantly impacted with gestational ethanol exposure (Varaschin et al., 2010, 2014). The mechanism behind the deficits in population spike potentiation are unknown but could be a result of dysregulation of the inhibitory tone (Everett et al., 2012; Kenton et al., 2020).

Choline supplementation from PND 10 to 30 did not result in long‐lasting alterations in synaptic plasticity for either control or ETOH offspring. This was surprising as previous work exploring the behavioral consequences of postnatal choline supplementation on learning and memory found supplemented animals had ameliorated deficits in the Morris Water Maze, a hippocampal‐dependent learning and memory task (Ryan et al., 2008; Thomas et al., 2007). Previous work from our laboratory also found postnatal choline supplementation improved LTP in the DG in early adolescence with a gestational ETOH exposure paradigm in rats (Grafe et al., 2022). There are several possible explanations for why choline did not have an effect in these experiments. The first is that there may exist subregional specificity and benefits of choline supplementation may be more evident in the CA1 region of the hippocampus. Additionally, supplemented choline may be utilized or stored quickly such that any positive benefits, which were present early in adolescence do not persist into adulthood. If this is the case, it is possible that continuing the choline treatment throughout the lifespan may extend the positive effects of choline on hippocampal synaptic plasticity. However, notably, the behavioral data from both preclinical studies and clinical studies suggest that the effects of early postnatal choline are long‐lasting (Gimbel et al., 2022). The final proposed explanation is that the positive long‐term effects are more subtle, such as in changes in LTP threshold (Pyapali et al., 1998), structural alterations in spine density (Meck et al., 2008), or epigenetic modulation (Otero et al., 2012; Zeisel, 2011). These hypotheses will require further study to uncover the long‐term effects of choline supplementation on the ETOH brain. It is also possible that the benefits of choline supplementation could be extended with additional treatments, such as exercise (Milbocker & Klintsova, 2020) or cognitive enrichment (Waddell et al., 2020).

Choline supplementation is being investigated as potential treatment for FASD with promising early results (Akison et al., 2018; Wozniak et al., 2015, 2020); clinical trials have shown improvements in working memory, IQ, and structural changes that sustained after a 4‐ and 7‐year follow‐up (Gimbel et al., 2022; Wozniak et al., 2015, 2020). These effects were limited to young children and were not evident when choline was supplemented later in childhood (Nguyen et al., 2016). Whether benefits of postnatal choline supplementation will persist into adulthood, as is seen in preclinical models, is yet unknown. As FASD is estimated to effect between 1% and 5% of the population (May et al., 2014), it is important to investigate long‐lasting therapies to improve cognitive outcomes. Our current findings indicate that deficits on some forms of hippocampal synaptic plasticity following the developmental ethanol exposure are evident into adulthood, but the positive influence of choline does not persist. Further work is required to determine other aspects of hippocampal function are altered with choline treatment, or if additional simultaneous treatments could extend the benefits of nutritional supplementation.

Taken together, the findings of the current study provide novel insight into the effect of developmental ETOH exposure on hippocampal synaptic plasticity. While there have been many studies investigating synaptic plasticity within animal models of FASD, methodological factors need to be taken into account when interpreting the results (Fontaine et al., 2016). Additionally, while choline supplementation is a promising method through which to alleviate some of the functional deficits imposed by developmental exposure to ETOH, the mechanism of action by which choline exerts its beneficial effects could be better elucidated. Evidence suggests that choline alters cholinergic development (Monk et al., 2012), phospholipid constitution (Bearer et al., 2015), and epigenetics (Otero et al., 2012), but how this translates to improved function is not well‐understood. Future studies that investigate the interplay between methodological differences (e.g., hippocampal subregion, timing of developmental ETOH exposure, and changes across postnatal development) will better enhance our understanding of how ETOH affects hippocampal synaptic plasticity in animal models of FASD.

## CONFLICT OF INTEREST STATEMENT

The authors of this work have no conflicts of interest to declare at this time.

## Supporting information


Table S1

## Data Availability

The data that support the findings of this study are available from the corresponding author upon reasonable request.
