# Supplementary material for: Developmental ethanol exposure produces deficits in long‐term potentiation in vivo that persist following postnatal choline supplementation
Source: Alcohol Clin Exp Res (Hoboken). 2024 Jun 8;48(8):1483–91. doi: 10.1111/acer.15384 (PMC11816008; doi:10.1111/acer.15384)
Supplement: Supplementary file 1 — Table S1 [file ACER-48-1483-s001.docx]

**SUPPLEMENTAL TABLE 1**

|  | Male PTP | Male LTP | Female PTP | Female LTP | Male:Female |
| --- | --- | --- | --- | --- | --- |
| CON+S | 37.8 ± 4.5 | 25.5 ± 3.7 | 35.9 ± 3.4 | 30.4 ± 2.6 | 11M:8F |
| CON+C | 34.3 ± 4.5 | 23.5 ± 1.9 | 35.7 ± 4.6 | 28.6 ± 4.3 | 11M:8F |
| ETOH+S | 29.0 ± 3.1 | 18.1 ± 2.5 | 27.4 ± 3.4 | 20.3 ± 2.9 | 7M:10F |
| ETOH+C | 25.6 ± 2.8 | 19.8 ± 2.6 | 30.2 ± 3.8 | 21.8 ± 5.1 | 9M:7F |

***Supplemental Table 1.*** Average male and female post-tetanic potentiation (PTP) and LTP ± SEM. Total animal number for male offspring (M) and female offspring (F).
